# Effects of exercise training on the biochemical pathways associated with sarcopenia

**DOI:** 10.20463/pan.2020.0019

**Published:** 2020-09-30

**Authors:** Dae Yun Seo, Boo Geun Hwang

**Affiliations:** 1 Cardiovascular and Metabolic Disease Center, Inje University, Busan, Republic of Korea; 2 Department of Sport Rehabilitation, Tong Myong University, Busan, Republic of Korea

**Keywords:** aerobic exercise, resistance exercise, sarcopenia, aging, skeletal muscle, IGF-1, PI3K, AKT, mTOR, TNF-α, Atrogin, MuRF1/2, ROS

## Abstract

**[Purpose]:**

Sarcopenia is considered one of the major causes of disability in the elderly population and is highly associated with aging. Exercise is an essential strategy for improving muscle health while aging and involves multiple metabolic and transcriptional adaptations. Although the beneficial effects of exercise modalities on skeletal muscle structure and function in aging are well recognized, the exact cellular and molecular mechanisms underlying the influence of exercise have not been fully elucidated.

**[Methods]:**

We summarize the biochemical pathways involved in the progression and pathogenesis of sarcopenia and describe the beneficial effects of exercise training on the relevant signaling pathways associated with sarcopenia.

**[Results]:**

This study briefly introduces current knowledge on the signaling pathways involved in the development of sarcopenia, effects of aerobic exercise on mitochondria-related parameters and mitochondrial function, and role of resistance exercise in the regulation of muscle protein synthesis against sarcopenia.

**[Conclusion]:**

This review suggested that the beneficial effects of exercise are still under-explored, and accelerated research will help develop better modalities for the prevention, management, and treatment of sarcopenia.

## INTRODUCTION

There is a growing body of literature that recognizes the impact of a decline in skeletal muscle mass and function associated with the progression of aging [1]. Aging is highly associated with an increased risk of developing sarcopenia [2]. Sarcopenia is a multifactorial disease that results in a gradual decline of skeletal muscle mass, strength, and functional performance [3]. Indeed, sarcopenia has proven to be a clinical relevance as a biomarker to identify the biological alterations associated with the progression of aging [4]. Despite the increasing evidence of the clinical implications of sarcopenia in the aging skeletal muscle, the exact mechanisms underlying of the sarcopenia are still unknown. It is crucial to determine the unbiased assessment methods for sarcopenia and include its diagnosis in routine clinical practice. Moreover, an incomplete pathological diagnosis of sarcopenia results in failed intervention strategies for clinical studies [5].

Physical inactivity might contribute to the development of sarcopenia [6]. Hence, current strategies for the management of sarcopenia include exercise training along with nutritional support [7]. Among the intervention strategies, exercise is well recognized to improve skeletal muscle function. Specifically, resistance exercise (RE) is a widely recommended strategy for the management of sarcopenia [8]. Recent studies have demonstrated that aerobic exercise (AE) inhibits the development of sarcopenia [9]. However, the cellular mechanisms through which RE and AE affect sarcopenia pathogenesis have not yet been determined.

Here, we briefly introduce currently available information on the signaling pathways involved in the development of sarcopenia. We also describe the beneficial effects of exercise on biochemical adaptations and discuss how this new paradigm provides a potential signal mechanism associated with the pathogenesis of sarcopenia.

### Insulin-like growth factor 1 (IGF-1)/Akt/mammalian target of rapamycin (mTOR)

Physiological maintenance of skeletal muscle tissue depends on the balance between anabolic and catabolic pathways [10]. The balance between protein synthesis and protein degradation is a decisive parameter for maintaining skeletal muscle mass during aging [11]. The activated phosphatidylinositol-3- kinase (PI3K)/Akt/mTOR signaling pathway plays a crucial role in anabolic metabolism induced by protein synthesis [12]. IGF-1 and insulin, which are representative anabolic stimuli, regulate the PI3K/Akt pathway, resulting in the control of skeletal muscle hypertrophy [13]. Increased IGF-1 and insulin levels inhibit the suppression of muscle synthesis by activating the phosphorylation of insulin receptor substrate 1 (IRS-1), resulting in the activation of the PI3K/Akt pathway and stimulation of mTOR [14]. Contraction-induced activation of mTOR depends on the phosphorylation of 70-kDa ribosomal S6 protein kinase (p70^S6K^) and 4E-binding protein 1 (4E-BP1), which promote protein synthesis [15]. Furthermore, protein kinase B (Akt) is essential for the regulation of cell metabolism [16].

### Forkhead Box O (FoxO) transcription factors

Skeletal muscle protein turnover influences the levels of insulin and IGF-1, which regulate skeletal muscle FoxO isoforms [17]. FOXO proteins, members of the Forkhead family of transcription factors, are identified by a conserved DNA-binding domain and act as key growth factors in the skeletal muscle. The FoxO family, comprising FoxO1, FoxO3, and FoxO4, regulates skeletal muscle metabolism [18]. These proteins are located in the nucleus and suppress the PI-3K/Akt and mTOR signaling pathways, resulting in a decrease in satellite cell number and regeneration [19,20]. FoxO1 is associated with anabolic pathways, which regulate phosphorylation of the translational repressor protein, 4E-BP1, and mTOR and regulatory-associated protein of mTOR (RAPTOR) [21]. The myonuclear levels of FoxO1 were found to be increased in the elderly compared to younger individuals [22].

### Transforming growth factor-beta (TGFβ)

Skeletal muscle atrophy can be negatively regulated by catabolic signals, a member of the TGFβ superfamily, regulating the expression of genes involved in myogenic differentiation and muscle regeneration [23]. Additionally, myostatin, which is produced by the skeletal muscle, regulates muscle growth [24]. The transcription factors of small mothers against decapentaplegic (SMAD) 2 and 3, which induce IGF-1/Akt signaling, regulate myostatin. Myostatin upregulates the ubiquitin ligases, atrogin1 and muscle RING-finger protein-1 (MuRF1), via FoxO transcription factors [25], causing muscle atrophy. The administration of myostatin inhibits the IGF1-PI3K-Akt pathway, which results in the activation of FoxO1 and increases the expression of atrogin-1 [24], whereas the inhibition of SMAD2/3 partially activates the mTOR signaling pathway that promotes skeletal muscle hypertrophy [26].

### Nuclear factor κB (NF-κB)

NF-κB is a protein complex and multifunctional regulator of DNA transcription, immune function, cell survival, and proliferation. Lower expression of myogenic differentiation 1 (MyoD) protein and MuRF1 seems to be caused by the activation of NF-κB during muscle atrophy [27]. It is well-known that NF-κB is activated in response to increased reactive oxygen species and tumor necrosis factor-alpha (TNF-α). IκB kinase is an enzyme complex associated with increased cellular inflammation and activated IkB results in a decrease in NF-κB in the cytosol [11]. Stimulation of IκB kinase induces IκBα phosphorylation, which regulates ubiquitination and subsequent proteolysis in the step of targeting IκBα, resulting in the binding of NF-κB [28].

### Mitogen-Activated Protein Kinases (MAPKs)

MAPKs, which are Ser/Thr kinases, regulate extracellular signals involved in a wide range of cellular processes, such as gene expression, apoptosis, and differentiation in eukaryotic cells [29]. Skeletal muscle myogenesis is controlled by four MAPK protein family members, including extracellular signal-regulated kinase (ERK) 1/2, p38 MAPK, c-Jun N-terminal kinases (JNKs), and ERK5. The upstream of MAPKs stimulates tyrosine and threonine residue phosphorylation after receiving signals from cytokines, growth factors, and cellular stressors, which results in the activation of MAPKs [30].

### Effects of exercise training on muscle pathophysiology

**Figure 1.**
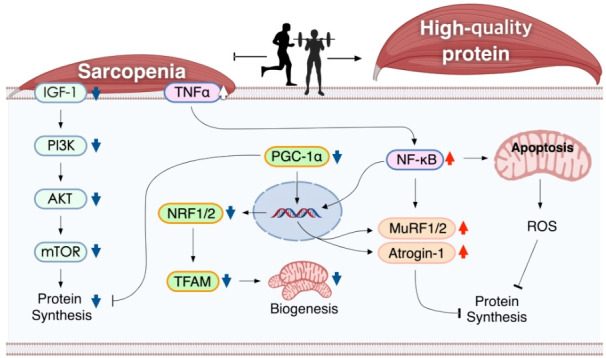
Mechanistic illustration of exercise-induced signaling pathways of the skeletal muscle in sarcopenia IGF-1, insulin-like growth factor-1; PI3K, phosphatidylinositol-3 kinase; AKT, protein kinase B; mTOR, mammalian target of rapamycin; TNF-α, tumor necrosis factor-alpha; PGC1-α, peroxisome proliferator-activated receptor gamma coactivator 1-alpha, NRF1/2: nuclear respiratory factor 1/2, TFAM: mitochondrial transcription factor A, MuRF1/2, muscle RING-finger protein-1, NF-kB: nuclear factor kappa-light-chain-enhancer of activated B, ROS: reactive oxygen species.

Physical inactivity and sedentary lifestyle are closely linked to a decline in muscle mass, physical fitness, and physical performance [31]. Conversely, exercise is one of the best strategies for improving the quality of life and health-related physical fitness factors and maintaining muscle mass and strength [32]. In general, physical exercise can be divided into AE, which involves low-intensity exercises for a long time, and RE, which involves powerful movements in a short time [33]. It is well known that both exercise regimes stimulate and regulate signaling pathways, such as the IGF-1/Akt/mTOR axis, FoxOs, NF-κB, MAPKs, mitochondrial function, and cell death in sarcopenia. The following paragraph summarizes the role of AE and RE in these pathways [34]. These exercises regulate the signaling pathways of the skeletal muscle in sarcopenia (Figure 1).

### AE

AE capacity contributes to the inhibition of aging [35]. It is also well known that AE improves cardiac and pulmonary function [36], which involves maximal oxygen uptake (VO_2_max); mitochondrial function [37], which involves mitochondrial density and activity; and energy metabolism [38], which involves insulin sensitivity and energy expenditure. Furthermore, AE inhibits intra-muscular lipid accumulation and aids in the recovery from muscle dysfunction in metabolic diseases [39]. AE also contributes to an increase in citrate synthase (CS) in muscle fibers, resulting in improved mitochondrial mass. Interestingly, acute and chronic AE contribute to the age-related decline of mitochondrial biogenesis, which is regulated by enzyme activities [40]. Table 1 summarizes the effect of AE on the signaling pathways of the skeletal muscle in sarcopenia.

To identify the beneficial effects of acute AE on mitochondrial functions of the skeletal muscle in the elderly, Cobley et al. studied the effects of acute AE on mitochondrial content, biogenesis, and mitochondria-related signaling pathways in sedentary elderly males. Their findings suggest that mitochondrial dysfunction and impairment of mitochondrial homeostasis with age-related decline in skeletal muscle are typically associated with physical inactivity. Interestingly, acute AE induced a significant increase in p38MAPK phosphorylation as well as PGC1-α and cyclooxygenase (COX)4 mRNA expression, suggesting a potential therapeutic effect of exercise on skeletal muscle adaptation to sustained skeletal muscle health in sedentary elderly individuals [41].

In contrast, chronic AE is widely considered to improve skeletal muscle function in the elderly. Broskey et al. examined the influence of exercise on mitochondrial function in older adults. They demonstrated that a 16-week AE program significantly increased the content of electron transport chain complexes, particularly that of complexes I, IV, and V in mitochondrial biogenesis (PGC1α and mitochondrial transcription factor A, TFAM), suggesting that chronic AE may protect muscles against aging by preventing mitochondrial and metabolic dysfunction [42]. Additionally, the oxidative phosphorylation capacity was greater in trained volunteers than sedentary volunteers. Following these findings, AE for 12 weeks stimulated mitochondrial biosynthesis and energy transport in aging rats [15,43]. AE also increased the levels of COX4 and Drp1. Finally, AE contributes to the activation of ATP synthase, which increases mitochondrial energy production [44]. AE results in an increase in PGC-1α signaling in the skeletal muscle [45], as demonstrated by an increase in PGC-1α levels in aged mice during the 12 weeks of AE program [43]. This mechanism is associated with a significant increase in TFAM, cytochrome c, and mtDNA content. AE also contributes to an increase in AMPK, p38MAPK, SIRT1, and p-cAMP response element-binding protein (CREB) levels. These results indicate that AE inhibits the reduction of aging-related factors and mitochondrial protein synthesis in the skeletal muscle [43]. In this regard, Broskey et al. observed an increase in electron transport complexes III, IV, and V in the skeletal muscle of elderly subjects during the 16 weeks AE program. Additionally, they reported a positive correlation between the expression of TFAM and PGC-1α.

AE also plays a role in regulating and inhibiting the development of apoptosis in aged skeletal muscle. Song et al. examined whether 12 weeks of AE reduced apoptotic DNA fragmentation and mitochondrial apoptotic signaling pathways in the skeletal muscle of aged rats. They reported the beneficial effect of AE on apoptotic signaling pathways [46]. Consistent with a similar study, AE also improved aging-induced apoptosis in the skeletal muscle of aged rats. Additionally, AE prevented the development of apoptotic signals by regulating the TNF-α signaling pathway in the extensor digitorum longus muscle of aging rats, resulting in improved exercise capacity and muscle strength [47]. Many researchers have attempted to elucidate the potential mechanisms of the beneficial effects of AE on skeletal metabolism in humans and have identified the role of IGF-1 [48]. Eight weeks of AE induced the upregulation of IGF-1, suggesting a correlation between VO_2_max and IGF-1 in males [49].

Interestingly, the basal levels of GH, IGF-1, and IGFBP-1 were higher in trained middle-aged men than sedentary individuals. Acute AE activated the GH/IGF1 axis in middle-aged men [50]. Sakamoto et al. found that acute submaximal and maximum intensity AE activated Akt r308 and Ser473 phosphorylation [51]. Similar to these results, Pasini et al. found that 8 weeks of AE increased the anabolic pathways in the skeletal muscle of aged rats. Additionally, AE resulted in an increased expression of insulin receptor and IRS-1 in the skeletal muscle of aged rats compared to sedentary rats [52].

### RE

RE is significantly effective in treating and preventing sarcopenia and acts by regulating muscle protein synthesis (MPS), thereby resulting in improved muscle strength and skeletal muscle mass [53]. Evidence suggests that acute and chronic RE play a crucial role in regulating skeletal muscle metabolism in elderly individuals [54]. However, the exact mechanism through which acute RE regulates skeletal muscle metabolism is not clearly known. Table 2 summarizes the effect of RE on the signaling pathways of the skeletal muscle in sarcopenia.

An acute bout of RE influences the intracellular mediators of MPS in the vastus lateralis muscle of young and older adults. This study observed an increase in the phosphorylation of mTOR, S6K1, 4E-BP1, and ERK1/2 in the younger group, while no such changes were seen in the older group. Moreover, a significant increase in MPS was observed in the younger group compared to the older group [55]. However, these findings contradict those reported by Raue et al., who examined whether acute RE affects several myogenic mediators at rest and after 4 h in young and older women. Acute RE stimulates the upregulation of MyoD and myogenic regulatory factor (MRF4) along with the downregulation of myostatin. The study also showed that high-intensity RE regulated the mRNA expression of ubiquitin proteasome-related genes and elevated atrogin-1 and MuRF-1 gene expression after 4 h, resulting in skeletal muscle atrophy in the vastus lateralis [56]. In contrast to these studies, Melov et al. showed that 6 months of RE in the elderly group resulted in a significant improvement in skeletal muscle strength.

Interestingly, the RE-induced elderly group had lower levels of senescence-related transcriptional genes than the young group. They also showed an improvement in mitochondrial function and muscle atrophy and a positive relationship between skeletal muscle phenotypes and the transcriptome [57]. In a similar study, Luo et al. showed that a 9-week RE program improved mitochondrial function, autophagy, and apoptosis in the gastrocnemius muscle of aged rats. These results suggest that RE is strongly associated with the reduction of 1A/1B-light chain 3 (LC3) -II/LC3-I ratio, p62 protein, Atgs, Beclin 1, Atg 5/12, Atg 7, and lysosomal enzyme cathepsin L in the skeletal muscle of aged rats, resulting in increased skeletal muscle mass [58].

It is known that skeletal muscle stimuli lead to the activation of AMPK phosphorylation and FoxO3A expression, resulting in the enhancement of skeletal muscle function and mass in the process of aging [59]. In particular, RE reduces the activation of cytochrome c and caspase-3, which is a representative inhibitor of apoptosis. In addition, RE induces IGF-1 activation and reduction of Akt and mTOR phosphorylation, resulting in improved antiapoptotic effects and inhibition of mitochondria-mediated apoptosis in aged skeletal muscle tissues [58]. Although RE modulates IGF-1 and its receptors as well as the Akt/mTOR and Akt/FoxO3a signaling pathways, the molecular mechanism underlying its role in protein synthesis is not fully understood. Additional studies are required to comprehensively examine the effect of RE on the IGF-1/Akt/mTOR and Akt/FoxO3a signaling pathways in the skeletal muscle.

## CONCLUSION

Sarcopenia is a multifactorial disease characterized by an age-related decline in skeletal muscle mass, strength, and function through the inhibition of protein synthesis and homeostasis. Exercise training, which regulates skeletal muscle metabolism, can promote sarcopenia prevention and delay. The benefits of exercise on sarcopenia are extensive and vary according to the exercise type, intensity, and frequency. This evidence suggests that chronic AE is recommended for enhanced maximal oxygen capacity and balanced skeletal muscle metabolism. Moreover, RE improves protein synthesis and inhibits protein degradation, resulting in the maintenance of skeletal muscle mass and improved skeletal muscle function in sarcopenia. Determining the concurrent effects of AE and RE in future studies will help clarify the pathogenesis of sarcopenia and its signaling pathways, thereby improving its treatment outcomes. Additionally, further research is needed to examine the effect of combined AE and RE on the signaling pathways of the skeletal muscle in sarcopenia.

## Figures and Tables

**Table 1.** Summarizes the effect of AE on the signaling pathways of the skeletal muscle in sarcopenia

Subjects	Types	Duration	Significance	Reference
Elderly males	Cycling exercise	Acute HIT	↑P38 MAPK/PGC1-α /COX3	Cobley et al. [41]
Older adults	Walking and biking	Chronic exercise (16 weeks)	↑ Electron transport chain complexes (I, IV, V)	Broskey et al. [42]
			↑ PGC1-α, TFAM	
Aged rats	Treadmill running	Chronic exercise (12 weeks)	↑ PGC1-α, TFAM, cytochrome c	Kang et al. [43]
			↑ P38 MAPK, AMPK, SIRT1	
Aged rats	Treadmill running	Chronic exercise (12 weeks)	↓ Mitochondrial apoptotic signaling pathways	Song et al. [46]
Aged rats	Treadmill running	Chronic exercise (4 weeks)	↓ TNF-α	Marzetti et al. [47]
			↑ Exercise capacity, muscle strength	
Older adults	Cycling exercise	Chronic exercise (8 weeks)	↑ IGF-1, VO2max	Poehlman et al. [49]
Aged rats	Treadmill running	Chronic exercise (8 weeks)	↑ mTOR, insulin receptor, IRS-1	Pasini et al. [52]

HIT: high-intensity training, P38 MAPK: p38 mitogen-activated protein kinases, PGC1-α: peroxisome proliferator-activated receptor gamma coactivator 1-alpha; COX3, cyclooxygenase; TFAM, mitochondrial transcription factor A; AMPK, AMP-activated protein

**Table 2.** Summarizes the effect of RE on the signaling pathways of the skeletal muscle in sarcopenia

Subjects	Types	Duration	Significance	Reference
Older adults	Leg extension (70% of 1RM)	Acute bout of RE	No significant mTOR, S6K1, 4E-BP1, ERK1/2	Fry et al. [55]
Old women	Knee extension (70% of 1RM)	Acute bout of RE	↑ MuRF-1, FOXO3A, atrogin-1	Rau et al. [56]
Older adults	Combined RE (80% of 1RM)	Chronic exercise (6 months)	↑ Skeletal muscle strength, mitochondrial function	Melov et al. [57]
			↓ Senescence-related transcriptional genes	
Aged rats	Ladder climbing (10 repetitions)	Chronic exercise (9 weeks)	↑ Skeletal muscle strength and mass, IGF-1	Luo et al. [58]
			↓ LC3-II/LC3-I ratio, p62	

1RM: one-repetition maximum; mTOR: mammalian target of rapamycin; S6K1: ribosomal S6 kinase1; 4E-BP1: eukaryotic initiation factor 4Ebinding protein1; ERK1/2: extracellular signal-regulated kinase 1/2; MuRF-1; muscle RING-finger protein-1; FOXO3A: forkhead box O3A; IGF-1: insulin-like growth factor-1; LC3: light chain 3
